# Dr. Fogo, on Certain Theories of Hydrocephalus

**Published:** 1810-01

**Authors:** A. Fogo

**Affiliations:** Newcastle


### *To the Editors of the Medical and Phyfical Journal.*

GENTLEMEN,

IN No. 129, there is a paper on Hydrocephalus; the writer hints that every man has a theory, and every man has a system. It would be fortunate if every man's theory and system were of the right kind. There are many theories, almost as many as there are practitioners, as well among what the writer contemptuously calls "Old Women Practitioners," as among the young, fashionable, consequential, affectedly cognoscent practitioners, who pretend to know a great deal more beyond their "bow and ridiculously affected grimace." It does not appear that the writer is more free of theory and system than his brethren. He considers all the diseases of children to be hydrocephalus, and uses much "torturing and twisting to persuade his readers that he has cured many, labouring under that (I believe always) fatal disease." He forms his diagnostic chiefly from the child passing black fæces, and considers every such child to have water in the head. If he had considered the circumstances with more attention, he might have been undeceived. The black stools have no more no more connection with water in the head than they have with water in the heel. Such black stools may be seen frequently from infancy to old age, where the liver has been in a state of chronic inflammation, obstruction, or congestion. In such cases, after a week or ten days course of an alterative medicine, such as is commonly given when the disease is discovered; the first symptom of recovery is the appearance of brown or black fæces, and of an unnatural smell. These are what an eminent physician calls "foul bowels," and another distinguishes by the name of "Hydrocephalic stools;" meaning, I suppose, that they are the effects of water contained in the brain! Such stools were brought down by the active purgatives of calomel and jalap; but all the purgatives he gave were incapable of extracting water out of the ventricles of the brain. The bowels were torpid from the deficiency of healthy bile, in consequence of the obstructed state of the biliary system, which was distinctly pointed out, when the black fæces made their appearance.

The writer complains, that the "cap blistering plasters" did not raise vesicles to his satisfaction. Where a blistering plaster is not wanted the vesicles are raised very slowly, but where there is any inflammatory action to be subdued, they rise very soon and give relief. This was probably the case where they raised vesicles slowly; it is possible they were not wanted.

I cannot harbour the idea that the writer means to impose on the credulity of his friends, by persuading them that every child who passes black fæces has water in the head; if he does, he will with the same facility persuade them, if the child recovers, that he has cured hydrocephalus, which, for reasons I will not trouble you with, I am afraid he never did, nor never will do.

Having made these few remarks, I will conclude with examining the result of the writers success in his practice.

| Case. No. | | |
|---|---|---|
| 1. | No black stools - | died. |
| 2. | Black stools - - | recovered. |
| 3. | Fœtid stools - - | recovered |
| 4. | Fœtid green stools | recovered. |
| 5. | No black stools - | died. |
| 6. | Black stools - - | recovered. |
| 7. | Dark coloured stools | died, head not opened. |
| 8. | Dark brown stools - | recovered. |
| 9. | No account of stools | died, head not opened. |
| 10. | No account of stools | died, head contained water and bloody water. |

Brown

*Brown Stools, black Fæces.*

| | | | |
|---|---|---|---|
| 11. | Jan. 22. | Addomen sore to the touch, | Recovered. |
| | 23. | Fœtid hard black stools, | |
| | 25. | Several black stools fœtid, | |
| | 26. | Stools natural, | |

There seems an impropriety in calling these black fæces "Hydrocephalic Stools." As in the above eleven cases, all such as had no black stools died, and all such as passed black fæces recovered, except No. 7, which case is of no use, as the head was not examined, consequently no proof of its being hydrocephalus.

I am, &c.

A. FOGO.

